# CCTA-derived plaque component-inflammation load is associated with concurrent CT-FFR-defined functional limitation: a retrospective cross-sectional imaging study

**DOI:** 10.3389/fcvm.2026.1913257

**Published:** 2026-09-11

**Authors:** Xiuxiu Hao, Baoqi Zhang, Lei Zhang, Yang Zhang

**Affiliations:** 1 Imaging Centre, Fuyang People’s Hospital, Fuyang, Anhui, China; 2 Imaging Centre, Fuyang People’s Hospital Affiliated to Anhui Medical University, Fuyang, Anhui, China

**Keywords:** cancer history, computed tomography fractional flow reserve, coronary computed tomography angiography, internal validation, pericoronary fat attenuation index, plaque composition

## Abstract

**Background:**

Coronary computed tomography angiography (CCTA) depicts coronary anatomy, plaque composition, and pericoronary inflammation, whereas CT-derived fractional flow reserve (CT-FFR) provides a computational functional estimate. We examined whether a prespecified plaque component-inflammation load (CIL) index was associated with concurrent CT-FFR-defined functional limitation.

**Methods:**

This single-center retrospective cross-sectional study included 334 patients (336 vessels or lesions) undergoing clinically indicated CCTA with quantitative plaque analysis, fat attenuation index (FAI) measurement, and CT-FFR computation. The CIL index was the equal-weight mean of standardized lipid component volume, fibrofatty component volume, lipid-to-fibrous ratio, and mean FAI. The primary patient-level endpoint was CT-FFR ≤0.80 in the lesion with the lowest CT-FFR value. Multivariable association, repeated stratified five-fold cross-validation, calibration, bootstrap optimism correction, and prespecified sensitivity analyses were performed.

**Results:**

Concurrent CT-FFR ≤0.80 was present in 119 patients (35.6%). A 1-standard-deviation higher CIL index was associated with higher odds of CT-FFR ≤0.80 (adjusted odds ratio 10.60, 95% confidence interval 5.09–22.10; *P* < 0.001). The clinical-plus-anatomy-plus-CIL model had an internally cross-validated AUC of 0.940, PR-AUC of 0.891, and Brier score of 0.090; its optimism-corrected AUC was 0.945. Sensitivity analyses were directionally consistent. Cancer-related subgroup and treatment-interaction estimates were exploratory and imprecise.

**Conclusion:**

The CIL index was strongly associated with concurrent CT-FFR status within this cohort. Because the index and endpoint were derived from the same CCTA examination, and no external validation, invasive physiological reference, or longitudinal outcomes were available, the results represent within-examination association and internal discrimination rather than independent diagnostic accuracy, prognosis, or established clinical utility.

## Introduction

Coronary artery disease assessment has progressively moved beyond a binary distinction between obstructive and non-obstructive stenosis. Evidence from stable coronary disease and contemporary imaging trials indicates that anatomical severity alone does not fully capture functional significance or subsequent cardiovascular risk, supporting integrated interpretation of anatomy, plaque characteristics, and physiology (1–3).

CT-FFR extends CCTA from anatomical assessment to a vessel-specific computational estimate of functional limitation. Randomized, diagnostic, and registry studies have shown that CT-FFR can reduce unnecessary invasive angiography and improve identification of physiologically important lesions beyond stenosis severity alone (4–7). Nevertheless, CT-FFR remains an image-derived estimate whose accuracy depends on image quality, coronary segmentation, anatomical modeling, and the post-processing environment.

CCTA also characterizes plaque composition and pericoronary inflammation. Low-attenuation non calcified plaque burden has been associated with myocardial infarction and acute coronary syndromes, while perivascular fat attenuation reflects local coronary inflammation and has been linked to residual cardiovascular risk and high-risk plaque biology (8–14). These domains may provide complementary information regarding the structural and inflammatory substrate accompanying functional coronary limitation.

Cancer and anticancer treatment may alter atherosclerotic and inflammatory biology (15–20). However, the present cohort was assembled from patients undergoing clinically indicated CCTA rather than from a dedicated cardio-oncology population. Cancer history was therefore treated as a prespecified clinical covariate, with cancer-stratified and treatment-related analyses limited to secondary exploratory evaluation.

We examined whether an equal-weight CIL index, constructed without CT-FFR information, was cross-sectionally associated with concurrent CT-FFR ≤0.80. The primary objective was estimation of the adjusted association. Internally cross-validated discrimination and calibration were secondary measures of within-cohort separation, while phenotype, cancer-related, and machine-learning analyses were exploratory.

## Materials and methods

### Study design and population

This retrospective cross-sectional imaging study included patients who underwent clinically indicated CCTA at Fuyang People's Hospital between November 2023 and August 2024. Cancer history or anticancer treatment was not an eligibility criterion. The study was approved by the Ethics Committee of Fuyang People's Hospital [approval number: (2025)45], and written informed consent was obtained.

Inclusion criteria were age ≥18 years; CCTA image quality sufficient for quantitative plaque analysis, FAI measurement, and CT-FFR computation; at least one focal atherosclerotic plaque in a major coronary branch; target-vessel reference diameter >2 mm with 30%–90% luminal stenosis; and availability of the clinical and imaging data required for analysis. Exclusion criteria were nondiagnostic CCTA, failed CT-FFR or FAI computation, unavailable quantitative plaque analysis, a target lesion outside the prespecified vessel-diameter or stenosis range, or missing essential clinical or imaging information.

The final dataset contained 334 unique patients and 336 analyzable vessels or lesions. For the primary patient-level analysis, the lesion with the lowest CT-FFR value in each patient was selected as the representative lesion. Lesion-level analysis with patient-clustered standard errors was prespecified as a sensitivity analysis.

### Analytic framework and scope of inference

All plaque components, FAI measurements, anatomical variables, and CT-FFR values were derived from the same CCTA examination. The estimand was therefore the cross-sectional association with concurrent CT-FFR status, not temporal prediction. CT-FFR was treated as a computational imaging classification rather than as an independent reference standard equivalent to invasive fractional flow reserve. Internal resampling quantified within-cohort stability and optimism but could not establish transportability, independent diagnostic validity, prognostic value, or clinical utility.

### CCTA-derived imaging variables

CCTA-derived variables included stenosis degree, minimum lumen area, maximum area stenosis, high-risk plaque features, total plaque volume, calcified and noncalcified plaque volumes, lipid, fibrofatty, and fibrous components, lipid-to-fibrous ratio, calcification and noncalcification fractions, mean and maximum FAI, and CT-FFR. Plaque components were obtained after coronary plaque segmentation and attenuation-based tissue classification. Mean FAI was used as the primary pericoronary inflammatory measure, and maximum FAI was evaluated in sensitivity analysis.

### CIL index calculation

The CIL index was prespecified before outcome modeling from four CCTA-derived variables: lipid component volume, fibrofatty component volume, lipid-to-fibrous ratio, and mean FAI. Each variable was standardized as Z(x) = [x-mean(x)]/standard deviation(x), and the index was calculated as [Z(lipid component)+Z(fibrofatty component)+Z(lipid-to-fibrous ratio)+Z(mean FAI)]/4. Each component therefore had an equal weight of 0.25. No regression coefficient, machine-learning importance score, or CT-FFR information was used to select or weight the components. Because less negative FAI values indicate higher attenuation, higher mean FAI contributed positively to the index.

For internal cross-validation, standardization parameters and any required preprocessing were estimated within each training fold and then applied to the corresponding held-out fold. Four component-inflammatory phenotypes (low burden, inflammation-dominant, component-dominant, and synchronous high burden) were retained as descriptive cross-classifications and were not optimized against CT-FFR.

### Exploratory cancer-related variables

Cancer-related variables included cancer history, anticancer treatment within the preceding 6 months, thoracic radiotherapy, and exposure to potentially cardiotoxic therapy. These variables did not define cohort entry. Cancer history was included in the main clinical adjustment set; stratified associations and treatment-interaction analyses were exploratory. Treatment categories were clinically heterogeneous and were not interpreted as a common causal exposure.

### Concurrent imaging endpoint

The endpoint was CT-FFR ≤0.80 in the representative lesion. The threshold was selected *a priori* because 0.80 is conventionally used to indicate physiologically important stenosis in fractional-flow-reserve-based assessment (4–7). In the present study, however, CT-FFR was computed from the same CCTA examination as the index components and was not an independent invasive physiological reference or a longitudinal clinical outcome.

### Statistical analysis

Continuous variables are reported as median (IQR) and categorical variables as number (percentage). Between-group comparisons used nonparametric or categorical tests as appropriate. HbA1c was summarized among available cases only and was not imputed or entered into the multivariable models.

The prespecified clinical model included age, sex, body mass index, hypertension, diabetes, dyslipidemia, smoking history, and cancer history. The clinical-plus-anatomy model additionally included stenosis degree and minimum lumen area. The primary model added the CIL index. A secondary complex comparator replaced the CIL index with its four individual components. The adjusted CIL association was reported per 1-standard-deviation increase as an odds ratio with 95% CI. Reduced covariate sets were used for cancer-stratified analyses to limit overfitting.

Internal discrimination was evaluated using averaged out-of-fold predictions from 50 repetitions of stratified five-fold cross-validation. AUC, PR-AUC, Brier score, calibration-in-the-large, and calibration slope were reported with 95% CIs; patient-level bootstrap resampling was used for metric intervals, and 1,000 full-pipeline bootstrap samples were used for optimism correction. Incremental changes after addition of CIL to the clinical-plus-anatomy model were estimated by paired stratified bootstrap. These analyses were internal validation only.

Sensitivity analyses evaluated additional high-risk plaque adjustment, stenosis <70%, exclusion of possible prior stent or percutaneous coronary intervention records, maximum FAI in place of mean FAI, an alternative anatomical adjustment, leave-one-component-out CIL indices, and lesion-level analysis with patient-clustered robust standard errors. Natural splines assessed nonlinearity. Cancer-related interactions were considered exploratory and were not interpreted as evidence of treatment-specific effects when confidence intervals were wide. XGBoost and fold-wise out-of-fold SHAP analyses were confined to the Supplementary Material as supportive descriptions of model behavior (21, 22). Reporting was informed by TRIPOD + AI, sample-size, and PROBAST principles while recognizing the cross-sectional, within-examination design (23–25). Analyses were performed in R version 4.5.3.

### Figure generation and reproducibility

**Figure 1:**
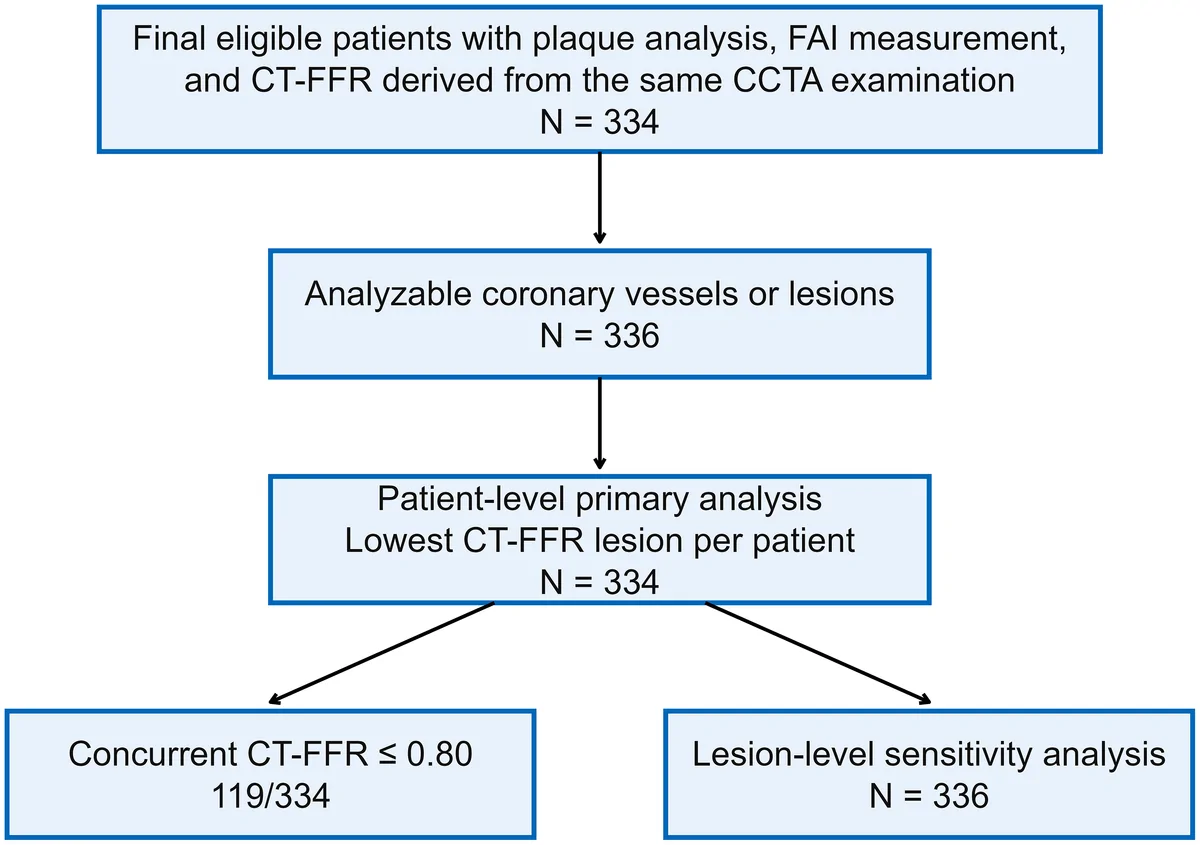
Analytic cohort structure. All plaque components, FAI measurements, and CT-FFR values were derived from the same CCTA examination. The primary patient-level analysis included 334 patients and used the lesion with the lowest CT-FFR value for each patient.

**Figure 2:**
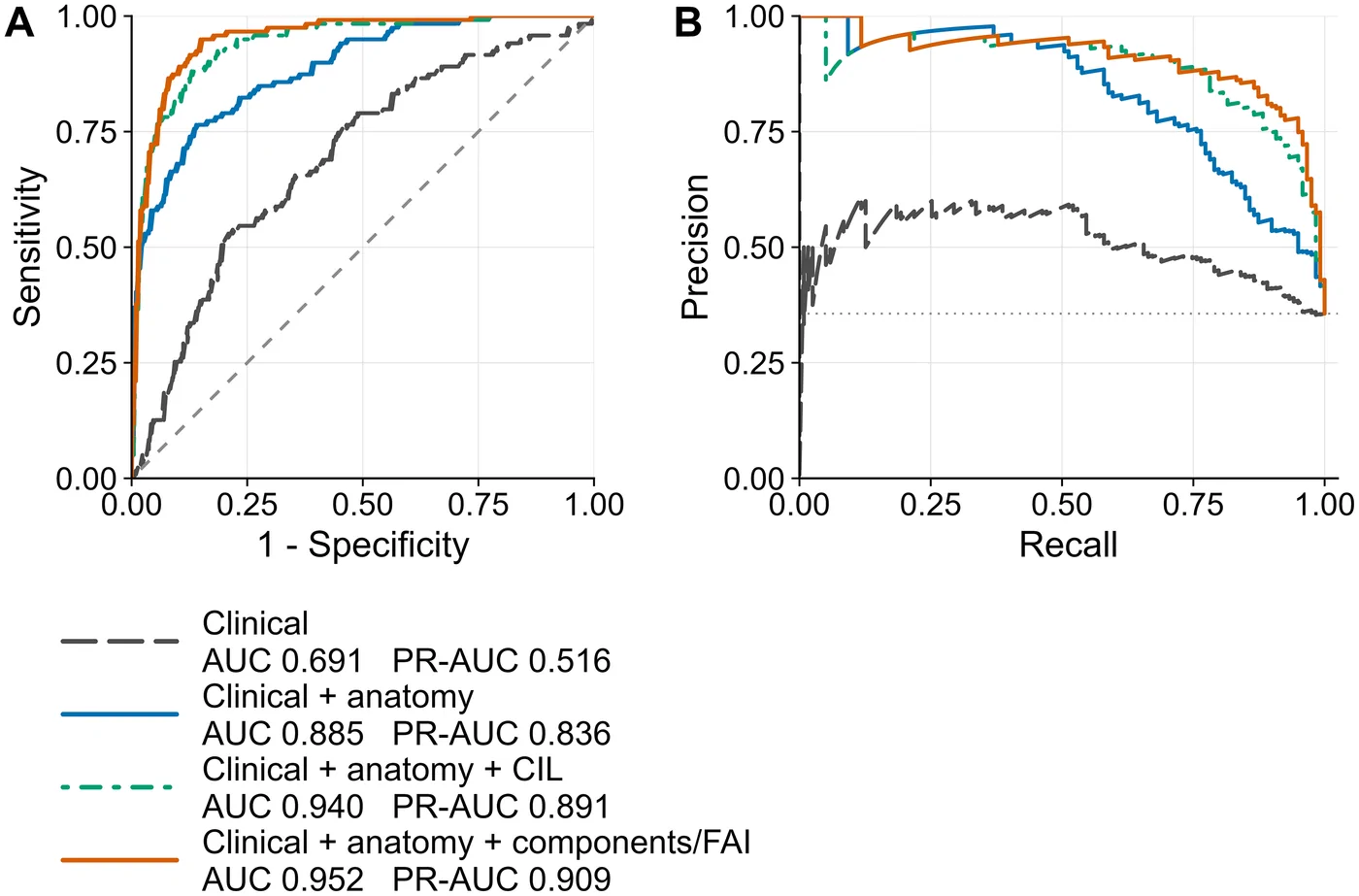
Discrimination of four prespecified comparison models for concurrent CT-FFR-defined functional limitation. **(A)** Receiver operating characteristic curves. **(B)** Precision-recall curves. Curves were generated from averaged out-of-fold predictions obtained through 50 repetitions of stratified five-fold internal cross-validation. The results do not constitute external validation or an independent diagnostic-accuracy assessment.

**Figure 3:**
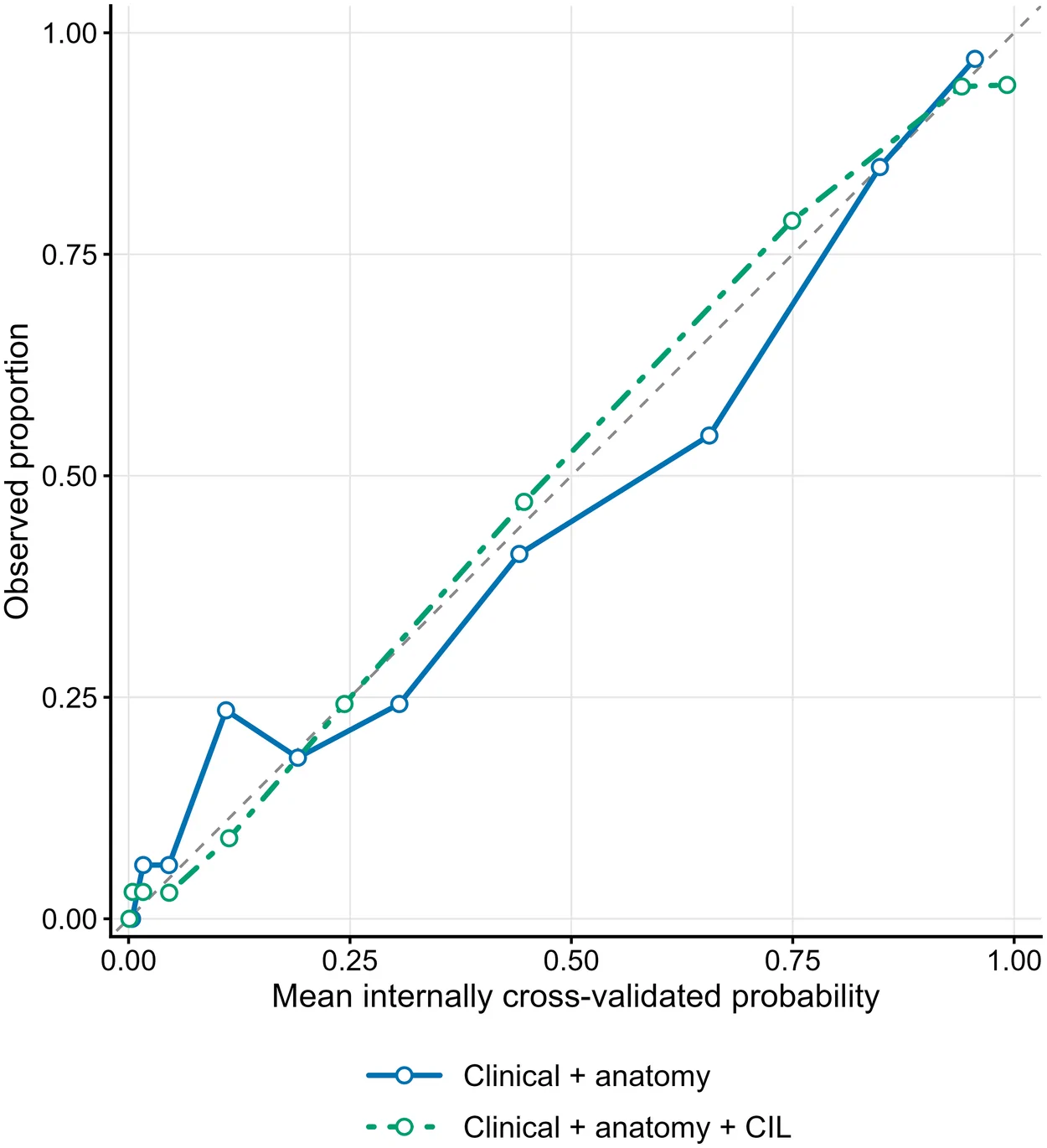
Internal calibration of the clinical-plus-anatomy model and the clinical-plus-anatomy-plus-CIL model using averaged out-of-fold predictions. The diagonal line represents perfect agreement within the present cohort.

**Figure 4:**
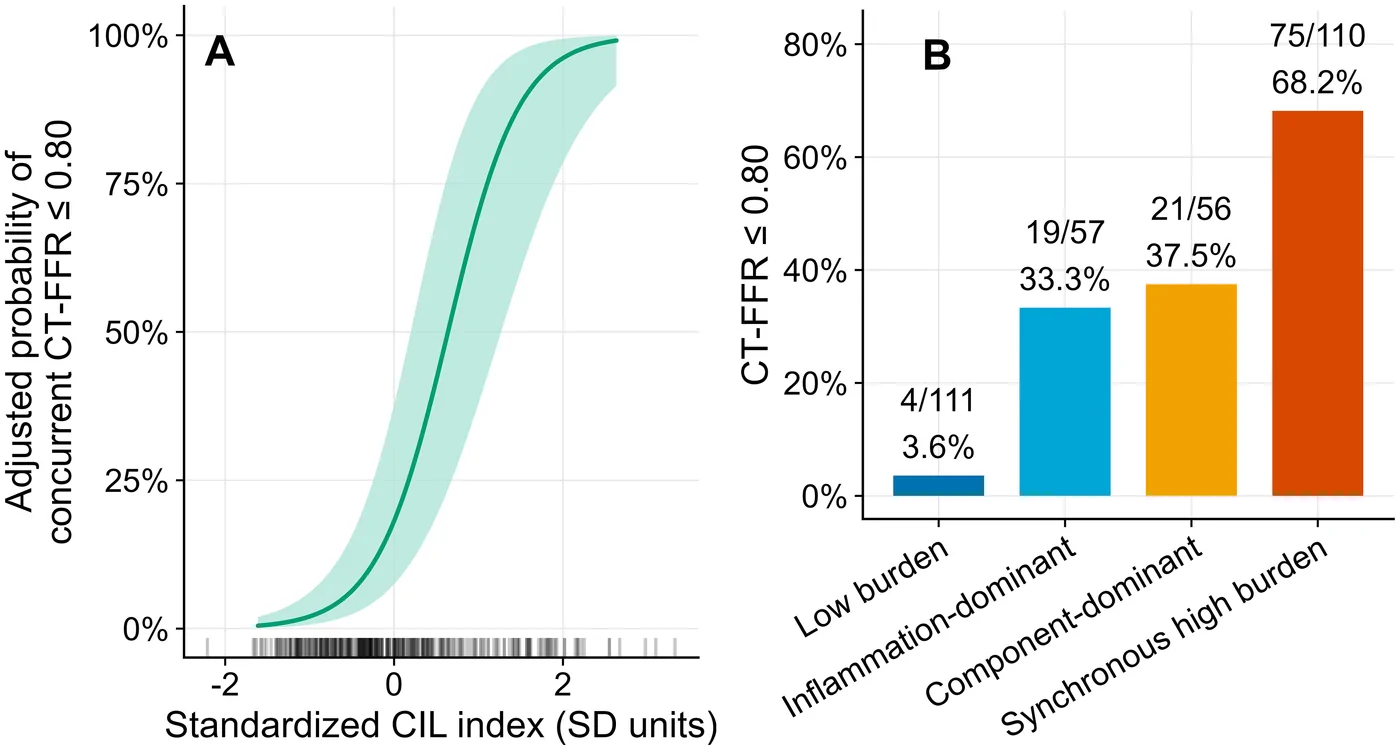
Adjusted association of the standardized CIL index with concurrent CT-FFR ≤0.80 and the descriptive gradient across component-inflammatory phenotypes. Panel **A** shows model-based probabilities with 95% CIs and rug marks for the observed CIL distribution; panel **B** shows observed proportions. Neither panel represents prospective risk prediction.

**Figure 5:**
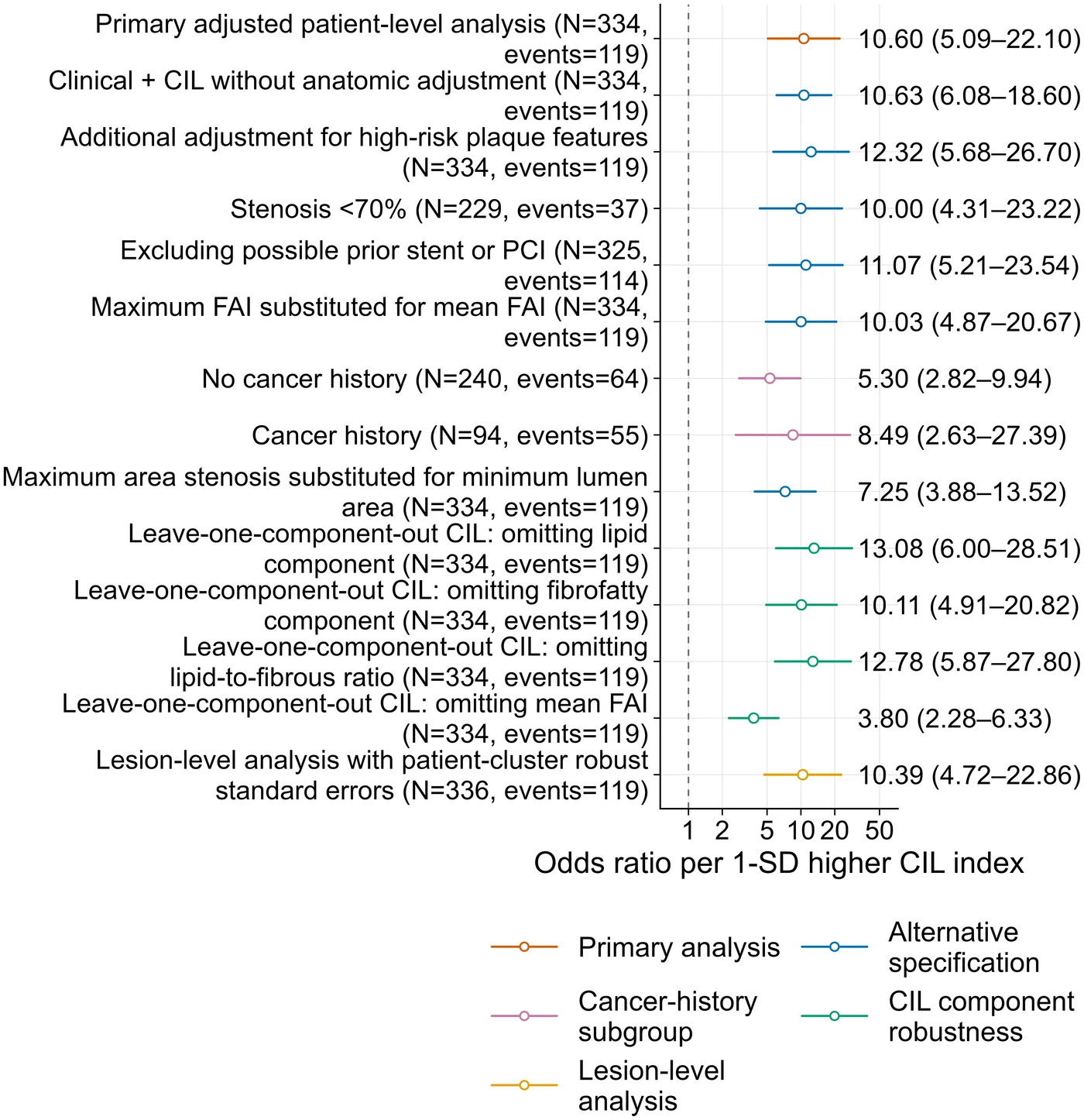
Primary and sensitivity analyses for the association of a 1-standard-deviation higher CIL index with concurrent CT-FFR-defined functional limitation. Cancer-stratified estimates are exploratory and should be interpreted with their confidence intervals.

All main and Supplementary Figures were generated in R version 4.5.3 from the same analysis datasets and numerical model outputs used for the reported results. Figure 1 was constructed from the finalized cohort accounting and shows the 334 unique patients, 336 analyzable vessels or lesions, selection of the lesion with the lowest CT-FFR value for the primary patient-level analysis, the 119 of 334 patients with concurrent CT-FFR ≤0.80, and the lesion-level sensitivity-analysis sample. Figure 2 presents receiver operating characteristic and precision-recall curves for the four prespecified comparison models; the curves were generated from averaged out-of-fold predicted probabilities obtained through 50 repetitions of stratified five-fold cross-validation, and AUC and PR-AUC were calculated from these same out-of-fold predictions. Figure 3 was generated from the same averaged out-of-fold predictions by plotting the mean predicted probability against the observed event proportion within the probability intervals used for the calibration display; the 45-degree line denotes perfect agreement. Figure 4A shows model-based adjusted probabilities with 95% confidence intervals across the observed standardized CIL range, with rug marks indicating the observed CIL distribution, whereas Figure 4B shows the directly observed proportions of CT-FFR ≤0.80 in the four prespecified component-inflammatory phenotypes. Figure 5 is a forest plot of the odds ratios per 1-standard-deviation increase in CIL and their 95% confidence intervals from the primary and prespecified sensitivity analyses.

Supplementary Figure 1 was generated from within-group phenotype counts according to cancer history. Supplementary Figure 2 displays the observed interaction odds ratios and 95% confidence intervals from the exploratory effect-modification models on a logarithmic odds-ratio scale. Supplementary Figure 3 was generated from fold-wise out-of-fold SHAP values from the exploratory XGBoost analysis; each participant's SHAP values were obtained from a model trained without that participant, with mean absolute SHAP values used for feature ranking and individual SHAP distributions displayed in the summary plot. For quality control, plotted cohort counts, event counts, performance metrics, effect estimates, and confidence intervals were cross-checked against the corresponding tabulated numerical results. No generative artificial intelligence, large language model, AI-assisted image-generation tool, or AI-assisted figure-editing tool was used for the statistical analyses or figure production. The only machine-learning component was the prespecified exploratory XGBoost/SHAP analysis in the Supplementary Material, for which the fold-wise out-of-fold procedure described above was used to limit information leakage and to assess internal model behavior.

## Results

### Study population

The final analysis included 334 patients and 336 analyzable vessels or lesions. Concurrent CT-FFR ≤0.80 was present in 119 patients (35.6%). Cancer history was documented in 94 patients (28.1%) and was treated as a secondary clinical characteristic rather than as the defining feature of the cohort. The analytic structure is shown in Figure 1, and baseline clinical and CCTA characteristics are summarized in Table 1.

**Table 1:** Baseline clinical and CCTA imaging characteristics according to concurrent CT-FFR status.

Variable	Overall	CT-FFR >0.80	CT-FFR ≤0.80	*P* value
Age, years	66.5 (57.0–74.0)	66.0 (56.5–74.0)	67.0 (58.0–73.0)	0.877
Female sex	164 (49.1%)	96 (44.7%)	68 (57.1%)	0.031
BMI, kg/m2	25.0 (22.2–27.3)	24.7 (22.1–27.2)	25.6 (22.7–27.4)	0.203
Hypertension	207 (62.0%)	127 (59.1%)	80 (67.2%)	0.159
Diabetes	92 (27.5%)	48 (22.3%)	44 (37.0%)	0.005
Dyslipidemia	153 (45.8%)	95 (44.2%)	58 (48.7%)	0.492
Smoking history	83 (24.9%)	57 (26.5%)	26 (21.8%)	0.359
Cancer history	94 (28.1%)	39 (18.1%)	55 (46.2%)	<0.001
Recent anticancer treatment	55 (16.5%)	15 (7.0%)	40 (33.6%)	<0.001
Thoracic radiotherapy	25 (7.5%)	10 (4.7%)	15 (12.6%)	0.015
Cardiotoxic therapy exposure	58 (17.4%)	20 (9.3%)	38 (31.9%)	<0.001
Stenosis degree, %	60.0 (49.0–73.0)	52.0 (42.5–63.5)	74.0 (66.5–82.5)	<0.001
Minimum lumen area, mm2	3.8 (2.2–5.0)	4.4 (3.4–5.4)	1.9 (1.0–3.3)	<0.001
Plaque volume	269.2 (217.0–329.7)	240.9 (195.2–289.4)	328.1 (278.4–372.1)	<0.001
Lipid component	36.7 (21.9–60.4)	27.1 (17.6–41.5)	69.3 (45.0–96.2)	<0.001
Fibrofatty component	90.6 (66.4–124.2)	77.0 (56.8–102.6)	118.2 (92.7–158.0)	<0.001
Lipid-to-fibrous ratio	1.3 (0.7–2.7)	1.0 (0.6–2.1)	2.5 (1.2–3.5)	<0.001
Mean FAI, HU	−79.5 (−83.7–74.3)	−81.5 (−85.5–78.5)	−73.2 (−78.4–68.4)	<0.001
Reconstructed CIL index, SD	−0.2 (−0.7–0.5)	−0.5 (−0.9–0.1)	0.7 (0.1–1.6)	<0.001

Data are median (IQR) or number (%). *P* values are descriptive. Cancer- and treatment-related descriptors were defined as described in the Methods.

### Baseline clinical and imaging characteristics

Patients with CT-FFR ≤0.80 more frequently had diabetes and female sex and had greater stenosis severity, smaller minimum lumen area, higher plaque burden, greater lipid and fibrofatty components, and higher mean FAI and CIL values than patients with CT-FFR >0.80 (Table 1). Cancer history and treatment-related descriptors were also more frequent in the CT-FFR ≤0.80 group.

### Primary association and internal discrimination

A 1-standard-deviation higher CIL index was associated with substantially higher odds of concurrent CT-FFR ≤0.80 after adjustment for age, sex, body mass index, hypertension, diabetes, dyslipidemia, smoking history, cancer history, stenosis degree, and minimum lumen area (odds ratio 10.60, 95% CI 5.09–22.10; *P* < 0.001). The bootstrap distribution of the association was directionally consistent.

Using averaged out-of-fold predictions, the clinical model had an AUC of 0.691, the clinical-plus-anatomy model an AUC of 0.885, the primary clinical-plus-anatomy-plus-CIL model an AUC of 0.940, and the secondary components-plus-FAI comparator an AUC of 0.952 (Figure 2 and Table 2). For the primary CIL model, PR-AUC was 0.891 and the Brier score was 0.090. Addition of CIL to the clinical-plus-anatomy model increased AUC by 0.055 (95% CI 0.030–0.083), increased PR-AUC by 0.054 (95% CI 0.018–0.092), and reduced the Brier score by 0.038 (95% CI 0.020–0.054). The optimism-corrected AUC, PR-AUC, and Brier score were 0.945, 0.902, and 0.088, respectively. These values quantify internal separation of a concurrent imaging endpoint and do not constitute external validation or independent diagnostic accuracy.

**Table 2:** Internal discrimination and calibration of the prespecified comparison models.

Model	AUC (95% CI)	PR-AUC (95% CI)	Brier (95% CI)	Calibration intercept (95% CI)	Calibration slope (95% CI)
Clinical	0.691 (0.631–0.749)	0.516 (0.453–0.599)	0.209 (0.192–0.226)	0.001 (−0.240 to 0.242)	0.794 (0.501–1.087)
Clinical + anatomy	0.885 (0.847–0.921)	0.836 (0.781–0.890)	0.127 (0.105–0.149)	−0.012 (−0.327 to 0.304)	0.869 (0.679–1.060)
Clinical + anatomy + CIL	0.940 (0.913–0.964)	0.891 (0.832–0.941)	0.090 (0.070–0.110)	0.009 (−0.363 to 0.381)	0.852 (0.663–1.041)
Clinical + anatomy + components and FAI	0.952 (0.927–0.973)	0.909 (0.858–0.951)	0.081 (0.061–0.102)	0.033 (−0.374 to 0.440)	0.794 (0.616–0.972)

All values are based on averaged out-of-fold predictions from repeated stratified five-fold cross-validation. AUC, PR-AUC, and Brier intervals were estimated by patient-level bootstrap. Calibration intercept and slope were obtained from logistic recalibration of the out-of-fold predictions. The components-and-FAI model was a secondary complex comparator.

### Internal calibration

Calibration based on averaged out-of-fold predictions is shown in Figure 3. For the primary CIL model, calibration-in-the-large was 0.009 (95% CI −0.363–0.381) and the calibration slope was 0.852 (95% CI 0.663–1.041). The corresponding values for the clinical-plus-anatomy model were −0.012 (95% CI −0.327–0.304) and 0.869 (95% CI 0.679–1.060). Calibration was therefore broadly compatible with the observed proportions within the present cohort, although deviations remained across probability intervals.

### CIL association and phenotype gradient

The adjusted probability of concurrent CT-FFR ≤0.80 increased progressively across higher standardized CIL values (Figure 4A). The overall CIL association was significant (*P* < 0.001), with no evidence of a nonlinear component (*P* = 0.726). CT-FFR ≤0.80 occurred in 4 of 111 patients (3.6%) with low burden, 19 of 57 (33.3%) with an inflammation-dominant phenotype, 21 of 56 (37.5%) with a component-dominant phenotype, and 75 of 110 (68.2%) with synchronous high burden (Figure 4B). These phenotypes are descriptive and require independent replication before classification use.

### Sensitivity and exploratory analyses

Results were directionally consistent across prespecified sensitivity analyses (Figure 5 and Supplementary Table 1). The odds ratio per 1-standard-deviation higher CIL was 10.00 (95% CI 4.31–23.22) among patients with stenosis <70%, 11.07 (95% CI 5.21–23.54) after exclusion of possible prior stent or percutaneous coronary intervention records, 10.03 (95% CI 4.87–20.67) when maximum FAI replaced mean FAI, and 10.39 (95% CI 4.72–22.86) in the lesion-level analysis. Omitting mean FAI attenuated but did not eliminate the association (odds ratio 3.80, 95% CI 2.28–6.33).

The association remained present in patients without cancer history (odds ratio 5.30, 95% CI 2.82–9.94) and in those with cancer history (odds ratio 8.49, 95% CI 2.63–27.39). The cancer-history interaction was not statistically significant (interaction odds ratio 0.51, 95% CI 0.14–1.84; *P* = 0.30), but treatment-specific interaction estimates were highly imprecise and did not exclude clinically important heterogeneity (Supplementary Table 2 and Supplementary Figure 2). The descriptive phenotype distribution by cancer history is shown in Supplementary Figure 1. Cross-validated SHAP results are shown in Supplementary Figure 3 and are not interpreted as causal effects.

## Discussion

In this single-center retrospective cross-sectional study, the equal-weight CIL index was strongly associated with concurrent CT-FFR ≤0.80 after adjustment for clinical and anatomical factors. Addition of the index improved internal discrimination and overall prediction error relative to the clinical-plus-anatomy model, and the association was consistent across multiple sensitivity analyses. These findings are best understood as within-examination concordance between plaque-inflammatory characteristics and a computational functional classification.

The biological rationale for combining plaque composition and pericoronary inflammation remains plausible. Stenosis describes lumen narrowing, whereas low-attenuation plaque components and perivascular fat attenuation characterize complementary structural and inflammatory features of coronary atherosclerosis (8–14). The CIL index summarized these domains using transparent, equal, prespecified weights rather than coefficients optimized against CT-FFR. The leave-one-component-out analyses also showed that no single plaque component fully accounted for the association, although the attenuation after omission of mean FAI suggests that the inflammatory domain contributed materially.

The central limitation is the lack of independence between the index and the endpoint. Plaque components, FAI, anatomical measurements, and CT-FFR were obtained from the same CCTA acquisition and may share image quality, coronary geometry, segmentation, motion, calcification, and post-processing influences. The high AUC values may therefore partly reflect shared imaging information. In addition, CT-FFR is a computational estimate rather than an invasive physiological reference. The study cannot be interpreted as an independent diagnostic-accuracy assessment.

Internal cross-validation, bootstrap confidence intervals, and optimism correction reduce concern about simple resubstitution bias, but they do not test transportability. The results may change with a different institution, scanner, reconstruction kernel, segmentation workflow, CT-FFR implementation, or patient spectrum. The reported calibration likewise describes the current cohort only. We therefore avoid interpreting the high internal discrimination as evidence of established clinical utility.

The absence of longitudinal outcomes further limits inference. CT-FFR ≤0.80 was measured concurrently and was not myocardial infarction, revascularization, cardiovascular death, or a major adverse cardiovascular event. The present analysis does not show that the CIL index predicts future outcomes or changes management. Prospective prognostic studies and diagnostic comparisons against invasive physiology address different questions and remain necessary.

Cancer-related findings were deliberately positioned as secondary. The cohort was not a dedicated cardio-oncology population, and most participants did not have a cancer history. Although the CIL association was observed in both cancer-history strata, the confidence interval in the cancer subgroup was wide, and treatment-defined interaction estimates were markedly imprecise. These data cannot establish cancer-specific risk identification, treatment-related coronary toxicity, or absence of effect modification (15–20). Dedicated cardio-oncology cohorts with detailed treatment class, dose, timing, cancer activity, and conventional cardiovascular risk characterization are required.

The exploratory XGBoost-SHAP analysis was restricted to the Supplementary Material. Fold-wise out-of-fold SHAP values showed that CIL, stenosis degree, minimum lumen area, and plaque volume contributed most strongly to the fitted model's internal behavior. SHAP values do not provide causal attribution, external validity, or evidence that a variable should guide clinical decisions, and they do not replace the prespecified logistic analysis (21, 22).

Study strengths include a clearly specified patient-level estimand, an index constructed without CT-FFR information, training-fold preprocessing during cross-validation, reporting of PR-AUC, Brier score, and calibration, paired assessment of incremental internal performance, and extensive sensitivity analyses. Limitations include the retrospective single-center design; selection of patients with analyzable plaque, FAI, and CT-FFR data; the same-examination origin of predictors and endpoint; absence of invasive physiological verification and external validation; limited quantification of calcification-related image degradation; incomplete HbA1c availability; small and heterogeneous cancer-treatment subgroups; and absence of longitudinal clinical outcomes.

Future studies should first evaluate technical reproducibility and transportability in independent multicenter cohorts using different scanners and post-processing environments. Studies designed for diagnostic accuracy should compare CIL with invasive physiology when clinically indicated, while prospective cohorts should assess whether it adds information beyond standard CCTA and CT-FFR for subsequent cardiovascular events. Cancer-specific questions should be investigated separately in adequately powered cardio-oncology cohorts.

## Conclusion

In a general clinically indicated CCTA cohort, an equal-weight CIL index was associated with concurrent CT-FFR-defined functional limitation and showed high internal discrimination. Because the index and endpoint were derived from the same examination, and neither external validation nor longitudinal outcomes were available, the findings are hypothesis-generating and do not establish independent diagnostic accuracy, prognosis, or clinical utility. Independent multicenter evaluation against invasive physiology and/or prospective cardiovascular outcomes is required before clinical application.

## Data Availability

The raw data supporting the conclusions of this article will be made available by the authors, without undue reservation.
