# Supplementary material for: CCTA-derived plaque component-inflammation load is associated with concurrent CT-FFR-defined functional limitation: a retrospective cross-sectional imaging study
Source: Front Cardiovasc Med. 2026 Sep 11;13:1913257. doi: 10.3389/fcvm.2026.1913257 (PMC13612346; doi:10.3389/fcvm.2026.1913257)
Supplement: Supplementary file 1 [file Datasheet1.docx]

**Supplementary Material**

This file reports prespecified sensitivity analyses, exploratory cancer-related analyses, additional internal-validation results, and fold-wise out-of-fold SHAP summaries. All imaging variables and CT-FFR values were derived from the same CCTA examination; the analyses do not constitute external validation, independent diagnostic accuracy, or longitudinal outcome assessment.

**Supplementary Table S1. Sensitivity analyses for the association between the CIL index and concurrent CT-FFR ≤0.80.**

| **Analysis** | **N** | **Events** | **OR per 1-SD** | **95% CI** | **Interpretation** |
| --- | --- | --- | --- | --- | --- |
| Primary adjusted patient-level analysis | 334 | 119 | 10.60 | 5.09-22.10 | Prespecified primary analysis |
| Clinical + CIL without anatomical adjustment | 334 | 119 | 10.63 | 6.08-18.60 | Assesses the association without direct anatomical adjustment |
| Additional adjustment for high-risk plaque features | 334 | 119 | 12.32 | 5.68-26.70 | Adds low-density plaque, napkin-ring sign, spotty calcification, and vulnerable plaque |
| Stenosis <70% | 229 | 37 | 10.00 | 4.31-23.22 | Limits dominance by severe anatomical stenosis |
| Excluding possible prior stent or PCI | 325 | 114 | 11.07 | 5.21-23.54 | Excludes 9 records identified by source-record text search |
| Maximum FAI substituted for mean FAI | 334 | 119 | 10.03 | 4.87-20.67 | Alternative inflammatory component |
| No cancer history | 240 | 64 | 5.30 | 2.82-9.94 | Exploratory stratified association |
| Cancer history | 94 | 55 | 8.49 | 2.63-27.39 | Exploratory stratified association |
| Maximum area stenosis substituted for minimum lumen area | 334 | 119 | 7.25 | 3.88-13.52 | Alternative anatomical adjustment |
| CIL omitting lipid component | 334 | 119 | 13.08 | 6.00-28.51 | Leave-one-component-out analysis |
| CIL omitting fibrofatty component | 334 | 119 | 10.11 | 4.91-20.82 | Leave-one-component-out analysis |
| CIL omitting lipid-to-fibrous ratio | 334 | 119 | 12.78 | 5.87-27.80 | Leave-one-component-out analysis |
| CIL omitting mean FAI | 334 | 119 | 3.80 | 2.28-6.33 | Leave-one-component-out analysis |
| Lesion-level analysis with patient-clustered robust standard errors | 336 | 119 | 10.39 | 4.72-22.86 | Two patients contributed more than one lesion |

The primary and sensitivity models used logistic regression. Cancer-stratified analyses used a reduced adjustment set. The lesion-level analysis used patient-clustered robust standard errors. OR, odds ratio; PCI, percutaneous coronary intervention.

**Supplementary Table S2. Exploratory cancer-related effect-modification analyses.**

| **Interaction** | **N** | **Events** | **Exposed N** | **Exposed events** | **Interaction OR** | **95% CI** | **P value** | **FDR P** |
| --- | --- | --- | --- | --- | --- | --- | --- | --- |
| Cancer history | 334 | 119 | 94 | 55 | 0.51 | 0.14-1.84 | 0.30 | 0.41 |
| Recent anticancer treatment among patients with cancer history | 94 | 55 | 55 | 40 | 1.49 | 0.19-11.95 | 0.71 | 0.71 |
| Thoracic radiotherapy among patients with cancer history | 94 | 55 | 25 | 15 | 13.15 | 0.55-316.82 | 0.11 | 0.26 |
| Cardiotoxic therapy exposure among patients with cancer history | 94 | 55 | 57 | 38 | 5.01 | 0.62-40.54 | 0.13 | 0.26 |

Interaction analyses were exploratory. Wide confidence intervals, particularly for treatment-defined subgroups, indicate substantial imprecision. A non-significant interaction should not be interpreted as proof of no clinically relevant heterogeneity.

**Supplementary Table S3A. Internal performance of the primary CIL model according to cancer history.**

| **Subgroup** | **N** | **Events** | **AUC (95% CI)** | **PR-AUC (95% CI)** | **Brier (95% CI)** |
| --- | --- | --- | --- | --- | --- |
| No cancer history | 240 | 64 | 0.932 (0.896-0.964) | 0.867 (0.805-0.922) | 0.086 (0.065-0.108) |
| Cancer history | 94 | 55 | 0.924 (0.859-0.977) | 0.923 (0.849-0.985) | 0.098 (0.057-0.143) |

Subgroup values were calculated from the same averaged out-of-fold predictions used in the full cohort and therefore represent internal subgroup performance only.

**Supplementary Table S3B. Bootstrap optimism correction for the primary CIL model.**

| **Metric** | **Apparent** | **Mean optimism** | **Optimism-corrected** |
| --- | --- | --- | --- |
| AUC | 0.954 | 0.009 | 0.945 |
| PR-AUC | 0.918 | 0.015 | 0.902 |
| Brier score | 0.078 | 0.010 | 0.088 |

Optimism correction used 1000 full-pipeline patient-level bootstrap samples.

**Supplementary Table S4. Association and nonlinearity tests for the standardized CIL index.**

| **Test** | **Comparison** | **P value** |
| --- | --- | --- |
| Overall association | Base covariate model versus natural-spline CIL model | <0.001 |
| Nonlinear component | Linear CIL model versus natural-spline CIL model | 0.726 |

The linear specification was retained because the nonlinear component was not statistically significant.

**Supplementary Figure S1. Descriptive distribution of component-inflammatory phenotypes according to cancer history.**

Percentages were calculated within each group. This comparison does not establish a causal effect of cancer or anticancer treatment.

**Supplementary Figure S2. Exploratory effect-modification analyses for cancer history and treatment-related exposures.**

Every finite interaction estimate is plotted at its observed odds ratio on a logarithmic scale. Very wide confidence intervals indicate substantial imprecision and do not support treatment-specific inference.

**Supplementary Figure S3. Exploratory cross-validated SHAP interpretation of a supportive XGBoost model.**

Each patient's SHAP values were obtained from a model trained without that patient. These values describe internal model behavior and do not establish causality, external transportability, independent diagnostic accuracy, or clinical utility.

**Supplementary abbreviations**

AUC, area under the receiver operating characteristic curve; CCTA, coronary computed tomography angiography; CI, confidence interval; CIL, component-inflammation load; CT-FFR, computed tomography fractional flow reserve; FAI, fat attenuation index; FDR, false discovery rate; OR, odds ratio; PR-AUC, area under the precision-recall curve; SHAP, SHapley Additive exPlanations; XGBoost, extreme gradient boosting.
